# Association between the fatty liver index and the risk of fracture among individuals over the age of 50 years: a nationwide population-based study

**DOI:** 10.3389/fendo.2023.1156996

**Published:** 2023-05-16

**Authors:** Goh Eun Chung, Eun Ju Cho, Min Joo Kim, Jeong-Ju Yoo, Yuri Cho, Kyu-na Lee, Kyungdo Han, Yoon Jun Kim, Jung-Hwan Yoon, Dong Wook Shin, Su Jong Yu

**Affiliations:** ^1^ Department of Internal Medicine and Healthcare Research Institute, Seoul National University Hospital Healthcare System Gangnam Center, Seoul, Republic of Korea; ^2^ Department of Internal Medicine and Liver Research Institute, Seoul National University College of Medicine, Seoul, Republic of Korea; ^3^ Department of Gastroenterology and Hepatology, Soonchunhyang University Bucheon Hospital, Bucheon, Gyeonggi-do, Republic of Korea; ^4^ Center for Liver and Pancreatobiliary Cancer, National Cancer Center, Goyang, Republic of Korea; ^5^ Department of Biomedicine & Health Science, Catholic University of Korea, Seoul, Republic of Korea; ^6^ Department of Statistics and Actuarial Science, Soongsil University, Seoul, Republic of Korea; ^7^ Department of Family Medicine/Supportive Care Center, Samsung Medical Center Supportive Care Center, Sungkyunkwan University School of Medicine, Seoul, Republic of Korea; ^8^ Department of Clinical Research Design and Evaluation/Department of Digital Health, Samsung Advanced Institute for Health Science, Seoul, Republic of Korea

**Keywords:** fracture, steatosis, osteoporotic, non-obese, fatty liver

## Abstract

**Background and purpose:**

The association between fatty liver and fracture risk has not been firmly established. In this study, we investigated the relationship between the fatty liver index (FLI) and the incidence of fractures among individuals ≥50 years of age, using a nationwide population-based cohort.

**Methods:**

Data from the Korean National Health Insurance System between January 2009 and December 2019 were analyzed using the Cox proportional hazards model. Fatty liver status was defined using FLI. Newly diagnosed fractures were identified based on insurance claim data.

**Results:**

Among the 3,384,457 individuals who met our inclusion criteria over the study period, 444,203 cases of incident fractures were identified over a median follow-up of 10.3 years. On multivariate analysis, the risk of fracture was significantly higher among individuals with a higher FLI score compared to those with an FLI<30, with adjusted hazard ratio [aHR] and 95% confidence interval [CI] as follows: FLI 30-59 group, aHR 1.04 and 95% CI 1.03-1.05; and FLI ≥60 group, aHR 1.12 and 95% CI 1.10–1.13. A higher FLI was associated with a greater risk of hip (aHR 1.23 and 1.52 for the FLI 30-59 and FLI ≥60 group, respectively) and vertebral fracture (aHR 1.08 and 1.16 for the FLI 30-59 and FLI≥60 group, respectively). The association between the risk for fracture and FLI ≥60 was prominent for non-obese than obese individuals (aHR 1.25 and 95% CI, 1.22–1.27 *versus* 1.06 and 1.05–1.08, respectively).

**Conclusions:**

A high FLI is associated with an increased risk of hip and vertebral fractures among individuals ≥50 years of age, suggestive of an association between a higher FLI and osteoporotic fractures.

## 1 Introduction

Osteoporosis-related fractures are an important cause of disability and mortality among postmenopausal women and older men. These fractures are associated with a high economic burden on health care systems and, thus, lowering the risk of these fractures has become a health priority due to the increasing life expectancy and aging of the general population (1). Low bone mineral density (BMD), which can lead to osteoporotic fractures, is associated with multiple risks factors, including insulin resistance, abdominal obesity, and metabolic syndrome, which itself has been linked to fatty liver disease (2–4).

Although an association between low BMD, osteoporosis, and fatty liver disease has previously been reported (5–8), the association between fatty liver and osteoporotic fractures remains controversial, with reported variation by age, sex, and obesity status. A previous study in Sweden has identified a slightly higher rate of osteoporotic fractures, and fractures overall, among patients with non-alcoholic fatty liver disease (NAFLD), although the long-term risk for fractures was comparable between patients with and without NAFLD (9). Meta-analysis studies have identified an association between NAFLD and osteoporotic fracture in men (10, 11), although these analyses are limited by the cross-sectional design of eligible studies. Recently, Kim et al. reported that a higher the fatty liver index (FLI) is associated with a higher risk of fracture (12). However, as the study included individuals ≥20 years of age, effects of female hormones and the skeletal site of fracture risk was not evaluated.

Non-obese fatty liver has been considered as a distinct subtype of fatty liver, characterized by different clinical features and metabolic risk factors (13, 14, 15). Accordingly, the association between fatty liver and the risk of fracture may differ depending on an individual’s obesity status. Thus, our aim in this study was to comprehensively investigate the association between the FLI and the risk of fracture among individuals ≥50 years, stratified by age, sex, and obesity status, using a nationwide Korean population-based database.

## 2 Material and methods

### 2.1 Data source

Data for this study was obtained from the Korean National Health Insurance System (NHIS) database. The NHIS is a national insurer managed by the Korean government, with approximately 97% of the Korean population subscribed (16). The NHIS database contains health records, including sociodemographic data (age, sex, and income level), medical diagnosis (based on the International Classification of Diseases, 10th revision [ICD-10]), treatment data, and health examination results (lifestyle and laboratory test results). The NHIS recommends that subscribers undergo a standardized medical examination, at least biennially (17).

### 2.2 Study sample

Eligible were the 10,601,283 individuals included in the NHIS in 2009. The exclusion criteria were as follows: age<50 years (n=6,130,272); diagnosis of liver cirrhosis (code K703, K746) or hepatitis (code B15-19) (n=609,556); missing data (n=185,246); and a previous history of fracture (n=291,752). After screening, the data of 3,384,457 participants were included in the analysis.

### 2.3 Ethics statement

The study protocol was approved by the Institutional Review Board of the Seoul National University Hospital (E-2007-135-1143) and conformed to the ethical guidelines of the World Medical Association Declaration of Helsinki. The requirement for informed consent from individuals was waived as de-identified secondary data were used in our analysis.

### 2.4 Covariates

Self-report questionnaires are used in the NHIS to collect relevant background data, as previously described (17, 18): age, sex, smoking status (non-smoker, ex-smoker, or current smoker), and alcohol consumption (none; mild, quantified as<30 g/day for males, and<20 g/day for females; and heavy, quantified as ≥30 g/day for males and ≥20 g/day for females). Regular physical exercise was quantified as >30 min of moderate physical activity ≥5 times/week or >20 min of high-intensity activity ≥3 times/week. Income level was dichotomized at the lowest 20%. Body mass index (BMI) was calculated as the weight (kg) divided by the square of the height (m) for each individual. Blood samples for analysis were obtained after an overnight fast ≥8 h.

The presence of health comorbidities and prescription drugs were identified based on the ICD-10 diagnosis codes, as follows: hypertension (codes I10–13 or I15) plus ≥1 prescription of antihypertensive agent or a systolic/diastolic blood pressure ≥140/90 mm Hg; diabetes, (code E11–14) plus ≥1 prescription of antidiabetic medication per year or a fasting glucose level ≥126 mg/dL; and dyslipidemia (code E78) plus ≥1 prescription of a lipid-lowering agent or a total cholesterol ≥240 mg/dL. Obesity was defined by a BMI ≥25 kg/m^2^, based on the Asia-Pacific World Health Organization criteria (19).

### 2.5 Measurement of fatty liver index

Although ultrasound imaging is considered as the first line of assessment for fatty liver disease (20), it is not included in the NHIS mass-screening program. Therefore, in our study, blood biochemistry data were used to identify the presence of fatty liver disease. The fatty liver index (FLI) score was calculated as previously described (21):


FLI=e0.953×In triglyceride+0.139×BMI+0.718×In GGT+0.053×WC-15.745/(1+e0.953×In triglyceride+0.139×BMI+0.718×In GGT+0.053×WC-15.745)×100,

where GGT is the level of gamma-glutamyl transferase and WC is the waist circumference. The FLI can range from ‘0’ to ‘100’, with a score<30 indicative of a low risk of fatty liver and a score ≥60 indicative of a high risk of fatty liver (22). For analysis, the study sample classified into the following three FLI scores:<30, 30–59, and ≥60.

### 2.6 Study outcome

The study population was followed from baseline to the date of diagnosis of a fracture or to the study endpoint of December 31, 2019, whichever occurred first. Participants who died during the follow-up period were censored at the time of death. The primary endpoint of this study was the incidence of fracture, defined using the ICD‐10 codes, as follows: hip (S72.0, S72.1, and S72.2), vertebral (S12.0, S12.1, S12.2, S22.0, S22.1, S32.0, M48.4, and M48.5), or other fractures. Other fractures identified included the clavicle (S42.0), upper arm (S42.2 and S42.3), wrist (S52.5 and S52.6), and ankle (S82.3, S82.5, and S82.6). A hip fracture was defined as one or more hospitalizations using the relevant diagnosis code. Vertebral and other fractures were defined as two or more outpatient visits, with an appropriate diagnosis code within a period of 12 months, as previously described (23, 24).

### 2.7 Statistical analyses

Data are presented as the mean ± standard deviation for normally distributed continuous variables, median and interquartil range for geometric means (95% confidence interval [CI]) for non-normally distributed continuous variables, and as proportions for categorical variables. Patient baseline characteristics between FLI groups were compared using independent t-tests and analysis of variance for continuous variables and the chi-squared test for categorical variables.

The primary outcome was the incidence rate of fractures, calculated by dividing the number of incident cases by the total follow-up period, reported per 1000 person-years. Cox proportional hazard regression was performed to estimate the risk of incident fracture. Multivariate analyses were adjusted for age, sex, BMI, income, smoking status, alcohol consumption, regular exercise, diabetes, hypertension, and dyslipidemia. A stratified subgroup analysis was conducted based on age (< 65 and ≥ 65 years), sex, obesity status, alcohol consumption (none to mild and heavy) and diabetes. Statistical analyses were performed using SAS version 9.4 (SAS Institute, Cary, NC, USA). Statistical significance was defined as a two-sided *P* value<0.05.

## 3 Results

### 3.1 Baseline characteristics of the study population

The median follow-up duration was 10.3 (interquartile range, 10.0-10.6) years, with 444,203 cases of incident fractures (13.1%) identified, including hip (n=32,621), vertebral (n= 176,642), and other (n= 234,940) fractures. The baseline characteristics of participants in each of the three FLI groups are shown in **Table 1**. Compared to the FLI<30 group, the FLI ≥60 group had a higher rate of males, current smokers, and heavy alcohol drinkers, as well as a higher proportion of individuals who exercised regularly and had higher income levels (*P*<0.001). Diabetes mellitus, hypertension, and dyslipidemia were also associated with a high FLI. Most anthropometric and laboratory variables (BMI, WC, fasting glucose, total cholesterol, high-density lipoprotein cholesterol, and triglycerides) were also less metabolically favorable in the FLI ≥60 than FLI<30 group (*P*<0.001).

**Table 1:** Baseline characteristics of participants according to fatty liver index.

	Fatty liver index			*P-value*
	< 30	30-59	≥ 60	
	(*n* =1,988,130)	(*n* =957,802)	(*n* =438,525)	
Age, years	60.4 ± 8.5	60.7 ± 8.0	59.5 ± 7.6	< 0.001
Male, *n* (%)	788,015 (39.6)	574,508 (60.0)	322,033 (73.4)	< 0.001
Smoking, *n* (%)				< 0.001
Non-smoker	1,453,618 (73.1)	561,157 (58.6)	208,122 (47.5)	
Ex-smoker	248,443 (12.5)	194,189 (20.3)	106,238 (24.2)	
Current smoker	286,069 (14.4)	202,456 (21.1)	124,165 (28.3)	
Alcohol consumption, *n* (%)				< 0.001
None	1,419,112 (71.4)	556,644 (58.1)	191,885 (43.8)	
Mild	494,477 (24.9)	322,441 (33.7)	173,225 (39.5)	
Heavy	74,541 (3.8)	78,717 (8.2)	73,415 (16.7)	
Regular exercise, *n* (%)	426,521 (21.5)	205,373 (21.4)	90,315 (20.6)	< 0.001
Income (lowest 20%), *n* (%)	441,119 (22.2)	201,560 (21.0)	95,199 (21.7)	< 0.001
Waist circumference (cm)	77.8 ± 6.5	86.5 ± 5.5	92.4 ± 6.8	< 0.001
Body mass index (kg/m^2^)	22.7 ± 2.3	25.4 ± 2.3	27.5 ± 3.0	< 0.001
Systolic blood pressure (mmHg)	124.0 ± 15.6	129.1 ± 15.4	132.2 ± 15.6	< 0.001
Diastolic blood pressure (mmHg)	76.4 ± 10.0	79.56 ± 9.9	81.8 ± 10.2	< 0.001
Comorbidity, *n* (%)				
Diabetes	194,044 (9.8)	174,205 (18.2)	119,228 (27.2)	< 0.001
Hypertension	716,867 (36.1)	510,217 (53.3)	278,075 (63.4)	< 0.001
Dyslipidemia	427,113 (21.5)	297,792 (31.1)	164,083 (37.4)	< 0.001
Laboratory findings				
Serum glucose (mg/dL)	97.9 ± 22.8	105.1 ± 28.9	113.1 ± 35.8	< 0.001
Total cholesterol (mg/dL)	198.4 ± 36.6	204.9 ± 38.7	209.9 ± 41.3	< 0.001
HDL-cholesterol (mg/dL)	57.7 ± 29.5	52.8 ± 31.6	51.2 ± 33.2	< 0.001
LDL-cholesterol (mg/dL)	120.9 ± 36.8	120.5 ± 40.4	111.2 ± 46.0	< 0.001
Triglyceride* (mg/dL)	95.4 (95.4-95.5)	152.9 (152.8-153.0)	222.4 (222.1-222.7)	< 0.001

Data are presented as mean ± standard deviation for continuous variables and n (%) for categorical variables.

HDL, high density lipoprotein; LDL, low density lipoprotein.

*Geometric means (95% confidence interval).

### 3.2 Association between the incident fracture rate and the fatty liver index

The fracture incidence rate varied as a function of the FLI classification, as follows: rate of 15.11 for the FLI<30 group; 13.02 for the FLI of 30-59 group; and 11.16 of the FLI ≥60 group. After adjusting for age, sex, BMI, income level, smoking status, alcohol consumption status, regular exercise, diabetes, hypertension, and dyslipidemia, the risk of fracture was significantly higher for higher FLI groups than the FLI<30 group (FLI<30-59, hazard ratio [HR], 1.04, and 95% CI, 1.03–1.05; and FLI ≥60, HR, 1.12, and 95% CI, 1.10–1.13). On analysis stratified by sex, the association between fracture risk and a FLI ≥60 was more prominent in males than females (males: aHR, 1.16 and 95% CI, 1.13–1.18; and females: aHR, 1.05 and 95% CI, 1.03–1.07; **Table 2**). When stratified by fracture site (hip, vertebral, or other), the association between higher FLI scores and incident fracture remained significant for hip and vertebral fractures (**Table 3**; hip fractures, FLI 30-59, aHR, 1.23 and 95% CI, 1.19–1.26, and FLI ≥60, aHR, 1.52 and 95%CI, 1.45–1.59; and vertebral fractures, FLI 30-59, aHR, 1.08 and 95% CI, 1.07–1.10, and FLI ≥60, aHR, 1.16 and 95%CI, 1.13–1.18). The association between fracture site and FLI classification remained significant for both males and females, with fracture risk at other sites also being greater for the high FLI group in females (**Supplementary Table 1**). When we performed sensitivity analysis in non-diabetic individuals, the stratification by FLI was still significant in non-diabetic population (**Supplementary Table 2**).

**Table 2:** Risk of incident fracture according to fatty liver index.

Fatty liver index	No. of population	No. of events	Follow-up duration (p-y)	Incidence rate (per 1,000 p-y)	Hazard Ratio (95% Confidence Interval)	
					Age and sex adjusted	Multivariate*
Total						
< 30	1,988,130	280,399	1,8553,619	15.11	1(Ref.)	1(Ref.)
30-59	957,802	117,529	9,023,694	13.02	0.98 (0.97,0.98)	1.04 (1.03,1.05)
≥ 60	438,525	46,275	4,144,778	11.16	1.02 (1.01,1.03)	1.12 (1.10,1.13)
*P* for trend					< 0.001	< 0.001
Male						
< 30	788,015	64,247	7,414,906	8.66	1(Ref.)	1(Ref.)
30-59	574,508	40,888	5,509,487	7.42	0.94 (0.93,0.95)	1.09 (1.07,1.10)
≥ 60	322,033	23,050	3,083,840	7.47	1.03 (1.01,1.04)	1.31 (1.28,1.34)
*P* for trend					< 0.001	< 0.001
Female						
< 30	1,200,115	216,152	11,138,713	19.41	1(Ref.)	1(Ref.)
30-59	383,294	76,641	3,514,207	21.81	1.00 (0.99,1.01)	1.03 (1.02,1.04)
≥ 60	116,492	23,225	1,060,939	21.89	1.01 (0.99,1.02)	1.06 (1.04,1.08)
*P* for trend					< 0.001	< 0.001

p-y, person-year;

*Adjusted for age, sex, body mass index, income, smoking, alcohol consumption, regular exercise, hypertension, diabetes, and dyslipidemia.

**Table 3:** Risk by fracture site according to fatty liver index.

Fracture site	Fatty liver index	No. of population	No. of events	Incidence rate (per 1,000 p-y)	Hazard Ratio (95% Confidence Interval)		
					Age and sex adjusted		Multivariate*
Hip	< 30	1,988,130	19,908	1.07	1(Ref.)	1(Ref.)	
	30-59	957,802	9,092	1.01	1.00 (0.97,1.02)	1.23 (1.19,1.26)	
	≥ 60	438,525	3,621	0.87	1.09 (1.05,1.13)	1.52 (1.45,1.59)	
	*P* for trend				< 0.001	< 0.001	
Vertebrae	< 30	1,988,130	109,233	5.88	1(Ref.)	1(Ref.)	
	30-59	957,802	48,886	5.42	1.02 (1.01,1.03)	1.08 (1.07,1.10)	
	≥ 60	438,525	18,523	4.47	1.04 (1.03,1.06)	1.16 (1.13,1.18)	
	*P* for trend				< 0.001	< 0.001	
Other	< 30	1,988,130	151,258	8.15	1(Ref.)	1(Ref.)	
	30-59	957,802	59,551	6.60	0.97 (0.96,0.98)	1.00 (0.99,1.01)	
	≥ 60	438,525	24,131	5.82	1.02 (1.01,1.03)	1.05 (1.03,1.07)	
	*P* for trend				< 0.001	< 0.001	

p-y, person-year.

*Adjusted for age, sex, body mass index, income, smoking, alcohol consumption, regular exercise, hypertension, diabetes, and dyslipidemia.

### 3.3 Subgroup analyses

**Figure 1:**
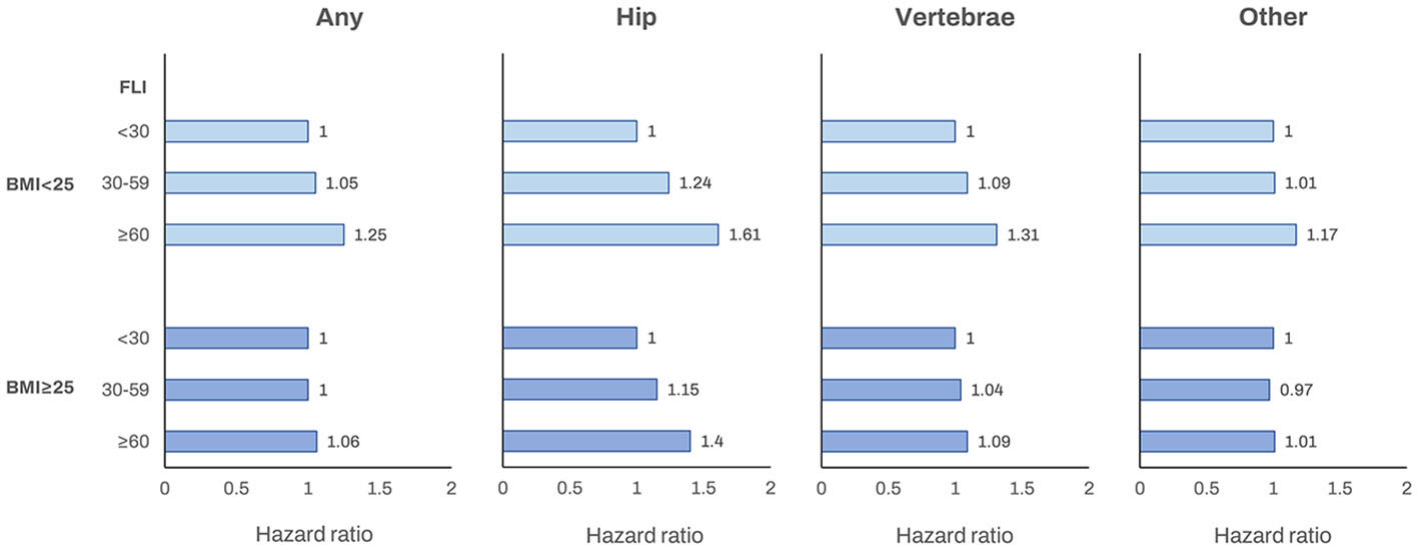
The association between the fatty liver index and incident fracture (any fracture, hip fracture, vertebrae fracture and other fracture) according to obesity status (BMI< 25 kg/m^2^ or ≥ 25 kg/m^2^). Adjusted for age, sex, income, smoking, alcohol consumption, regular exercise, hypertension, diabetes, and dyslipidemia. BMI, body mass index; FLI, fatty liver index.

Subgroup analyses were performed for age (< 65 vs. ≥ 65 years), obesity status (BMI ≥ 25 kg/m^2^ vs. < 25 kg/m^2^), alcohol consumption (none to mild vs. heavy) and diabetes. For the FLI ≥ 60 group, the increased risk of fracture was higher for younger (< 65 years), non-obese, none-to mild drinker, and non-diabetes groups (all *P* for interaction< 0.001; **Table 4**). The order of increased risk of fracture site in non-obese patients was hip, followed by vertebral and other fractures (**Figure 1**). With further stratification by sex, the increased risk of fracture in non-obese patients remained significant in both males and females (**Supplementary Table 3**).

**Table 4:** Stratified analyses of the association between fatty liver index and incident fracture: Hazard ratio (95% Confidence Interval) for incident fracture.

Stratified variables	Category	Fatty liver index			
		< 30	30-59	≥ 60	*P* for interaction
Age (years)	< 65	1 (Ref.)	1.08 (1.07,1.09)	1.18 (1.67,1.20)	< 0.001
	≥ 65	1 (Ref.)	1.00 (0.99,1.01)	1.04 (1.02,1.06)	
Body mass index (kg/m^2^)	< 25	1 (Ref.)	1.05 (1.04,1.06)	1.25 (1.22,1.27)	< 0.001
	≥ 25	1 (Ref.)	1.00 (0.99,1.02)	1.06 (1.05,1.08)	
Alcohol consumption	None to mild	1 (Ref.)	1.04 (1.03,1.05)	1.12 (1.10,1.13)	< 0.001
	Heavy	1 (Ref.)	0.97 (0.94,1.01)	1.09 (1.06,1.13)	
Diabetes	No	1 (Ref.)	1.05 (1.04,1.06)	1.13 (1.11,1.14)	< 0.001
	Yes	1 (Ref.)	1.00 (0.98,1.02)	1.08 (1.06,1.10)	

Adjusted for age, sex, body mass index, income, smoking, alcohol consumption, regular exercise, hypertension, diabetes, and dyslipidemia.

## 4 Discussion

In this study, we found that FLI was significantly associated with the risk of fracture in most Korean adults aged > 50 years. Among the fracture sites, the risk for fracture with a higher FLI was highest for hip fractures, followed by vertebral fractures, suggestive of an association between FLI and osteoporotic fractures rather than trauma-related fractures. The higher risk of fractures in the FLI ≥60 group was more prominent in males than females, as well as among individuals<65 years of age, and those who were non-obese. Therefore, the association between fatty liver and fracture risk is modified by sex, age, and obesity. The strengths of our study include the use of nearly the entire Korean population ≥50 years of age, which allows for generalizability of our findings and for the use of the hard endpoint of fracture, with nearly complete results of all skeletal sites. Furthermore, the inclusion of a large-scale nationwide cohort permitted stratified analyses using multiple variables.

The mechanisms underlying the association between FLI and fractures have not yet been fully elucidated. It is known that hepatic steatosis induces chronic, low-grade inflammation and insulin resistance, which may be involved in the development of BMD loss and osteoporosis, leading to a higher risk of fracture. Previous studies have provided support of an involvement of inflammatory cytokines, such as interleukin-6 and tumor necrosis factor, in the pathogenesis of osteoporosis (25, 26). Accumulation of intrahepatic lipids in patients with fatty liver induces insulin resistance (27) which may be associated with lower BMD and decreased strength of the femoral neck (28, 29). In addition, an *in vitro* study showed that insulin resistance in bone cells causes reduced bone strength and increased osteoporosity (30). Bone mass is maintained by the continuous formation and resorption of bone; as such, a disequilibrium of these processes might lead to changes in bone mass. The regulation of bone turnover is disturbed in patients with NAFLD and diabetes, suggesting that fatty liver is associated with an increased risk of altered bone turnover and fractures (31).

Among all fracture sites, hip fractures are regarded as the most severe of osteoporotic fractures, requiring hospitalization as well as being associated with high morbidity and low quality of life (32). In our study, the highest aHR associated with a high FLI was for hip fractures, followed by vertebral fractures. The higher risk for hip than other types of fractures is consistent with findings from a previous study, in which NAFLD in men was associated with a higher risk of hip fractures, although there was no statistical significance (33). Of note, this was a cross-sectional, observational study with a small sample size which may have made it difficult to show statistical significance. Although the specific link between hip fractures and FLI has not been clearly elucidated, various factors related to fatty liver may influence the complex pathogenesis of hip fractures. Abdominal obesity, a major risk factor for fatty liver, has been associated with a higher risk of hip fracture in a previous meta-analysis (34), resulting from the adverse effects of abdominal obesity–related inflammation on bone strength and, thus, fracture risk (35). In addition, sarcopenia, which has a significant positive association to NAFLD, increases the risk of hip fracture (36). With regard to vertebral fractures, as obesity is positively associated with trabecular and cortical bone parameters and negatively associated with cortical volumetric BMD (37), obesity may reduce vertebral fractures somewhat, providing an explanation for the more pronounced association between a higher FLI and hip than vertebral fractures.

Although the association between fatty liver and low BMD is influenced by sex and skeletal site of fractures, previous studies did not include complete results of all skeletal sites when evaluating sex-specific differences and, thus, results have remained inconclusive. Two studies from China reported that the presence of NAFLD increased the risk of osteoporotic fractures in men but not women (38, 33). A recent meta-analysis reported that the risk of osteoporosis or osteoporotic fracture was higher in men with than without NAFLD, with no significant difference among women with and without NAFLD, after adjusting for confounders (11). In our study, the association between FLI and fracture was significant in both sex but with higher aHRs for men than women. These findings may be related to sex-specific differences in bone structure and strength, body fat deposition, and hormones, which play an important role in bone remodeling by promoting bone formation and inhibiting bone breakdown (38, 39).

The relationship between obesity and fracture risk is known to be complex, with obesity having a divergent influence on fracture risks across different skeletal sites (40). Previous epidemiological studies reported a lower risk of fractures among individuals with obesity, while the risk increased for some fractures (ankle, femur, and humerus) and decreased for others (hip and wrist) with overweight and obesity (41). The stratified analysis by obesity in our study revealed that the risk of incident fracture in the FLI ≥60 group was higher for non-obese than obese individuals (aHR, 1.25 *vs* 1.06, respectively), with similar results observed in each fracture site. These findings suggest a strong association between the FLI and fracture risk in non-obese individuals and might be related to the protective effects of obesity on the risk of fracture.

This study had some limitations which should be acknowledged. First, the nature of fractures reported may be heterogeneous as fractures resulting from extrinsic trauma or traffic accidents could not be excluded owing to the unavailability of information on the causes of fracture in the NHIS database. We do note, however, that high-energy traumatic fractures are prominent in younger than older individuals and in men (42). Second, although liver biopsy is considered as the gold standard to diagnosis fatty liver, we used FLI as a surrogate marker of fatty liver. It has been previously shown that the FLI, as a surrogate marker for fatty liver, accurately diagnoses steatosis and correlates with insulin resistance (43). Third, the population in this study included all alcohol consumption habits; therefore, we could not limit the results to NAFLD or alcoholic fatty liver. However, we performed a stratified analysis by alcohol consumption and found that the association between FLI and fracture was more prominent in none to mild drinkers than in heavy drinkers. Fourth, although a previous history of fracture is an important risk factor for future fracture, we could not perform analysis in patients with earlier history of fractures. Due to the nature of the claim data, once a fracture is diagnosed, a diagnosis code remains at follow-up, making it difficult to define a new fracture event. In addition, we could not obtain further information on factors known to influence fracture risk, including bone markers, intake of vitamin D or calcium, and muscle strength (33). Also, we could not correct for some known determinants of fragility fracture risk such as parenteral history of fragility fracture and other disease or medications that make the bone weak. Thus, the association between FLI and fragility fracture has yet to be confirmed, and further studies are needed.

## 5 Conclusions

A high FLI score was associated with an increased risk of incident hip and vertebral fractures. These findings imply an association between FLI and osteoporotic fractures. Future studies are needed to investigate the mechanism underlying the association between FLI and osteoporotic fractures.

## Data availability statement

The datasets presented in this article are not readily available because The dataset (NHIS-HEALS) supporting the conclusions of this article is available in the homepage of National Health Insurance Sharing Service. To gain access to the data, a completed application form, a research proposal, and the applicant’s approval document from the institutional review board should be submitted to and reviewed by the inquiry committee of research support in NHIS. Currently, the use of NHIS data is allowed only for Korean researchers. Requests to access the datasets should be directed to http://nhiss.nhis.or.kr/bd/ab/bdaba021eng.do.

## Ethics statement

The studies involving human participants were reviewed and approved by Seoul National University Hospital. Written informed consent for participation was not required for this study in accordance with the national legislation and the institutional requirements.

## Author contributions

GC and EC drafted and revised the manuscript. J-JY, MK, and YC collected and reviewed the data, and revised the manuscript. K-nL and KH performed statistical analysis and revised the manuscript. DS and SY conceived the research idea, determined the study design, collected data, and revised the manuscript. All the authors have reviewed the manuscript.
