# Supplementary material for: Association between the fatty liver index and the risk of fracture among individuals over the age of 50 years: a nationwide population-based study
Source: Front Endocrinol (Lausanne). 2023 May 16;14:1156996. doi: 10.3389/fendo.2023.1156996 (PMC10227615; doi:10.3389/fendo.2023.1156996)
Supplement: Supplementary file 1 [file Table_1.docx]

**Supplementary Table 1. Risk by fracture site according to fatty liver index stratified by sex.**

| Fracture site | FLI | Male | | Female | | |
| --- | --- | --- | --- | --- | --- | --- |
|  |  | IR (1,000 p-y) | HR (95% CI) | | IR (1,000 p-y) | HR (95% CI) |
| Hip | 0-30 | 1.02 | 1(Ref.) | | 1.11 | 1(Ref.) |
|  | 30-59 | 0.68 | 1.26 (1.20,1.32) | | 1.51 | 1.22 (1.17,1.26) |
|  | ≥ 60 | 0.58 | 1.67 (1.56,1.78) | | 1.72 | 1.51 (1.42,1.60) |
|  | *P* for trend |  | < 0.001 | |  | < 0.001 |
| Vertebrae | 0-30 | 3.78 | 1(Ref.) | | 7.29 | 1(Ref.) |
|  | 30-59 | 3.04 | 1.10 (1.08,1.13) | | 9.15 | 1.08 (1.07,1.10) |
|  | ≥ 60 | 2.84 | 1.32 (1.28,1.36) | | 9.19 | 1.11 (1.08,1.14) |
|  | *P* for trend |  | < 0.001 | |  | < 0.001 |
| Other | 0-30 | 3.86 | 1(Ref.) | | 11.01 | 1(Ref.) |
|  | 30-59 | 3.70 | 1.05 (1.03,1.07) | | 11.14 | 0.98 (0.97,1.00) |
|  | ≥ 60 | 4.05 | 1.22 (1.18,1.25) | | 10.97 | 0.97 (0.95,0.99) |
|  | *P* for trend |  | < 0.001 | |  | 0.007 |

Abbreviations: FLI, fatty liver index; p-y, person-year; IR incidence rate; HR, hazard ratio; CI, confidence interval

*Adjusted for age, body mass index, income, smoking, alcohol consumption, regular exercise, hypertension, diabetes, and dyslipidemia

**Supplementary Table 2. Risk of incident fracture according to fatty liver index in non-diabetic individuals.**

| Fatty liver index | No. of population | No. of events | Follow-up duration  (p-y) | Incidence rate  (per 1,000 p-y) | HR (95% CI) |  |
| --- | --- | --- | --- | --- | --- | --- |
|  |  |  |  |  | Multivariate* | |
| Total |  |  |  |  |  | |
| < 30 | 1,794,086 | 249,056 | 16,835,051 | 14.79 | 1 (Ref.) | |
| 30-59 | 783,597 | 93,575 | 7,436,261 | 12.58 | 1.05 (1.04, 1.06) | |
| ≥ 60 | 319,297 | 32,356 | 3,045,347 | 10.62 | 1.13 (1.11, 1.14) | |
| Male |  |  |  |  |  | |
| < 30 | 693,191 | 55,622 | 6,564,965 | 8.47 | 1(Ref.) | |
| 30-59 | 469,554 | 32,770 | 4,534,619 | 7.23 | 1.10 (1.08, 1.12) | |
| ≥ 60 | 236,615 | 16,575 | 2,284,124 | 7.26 | 1.33 (1.30, 1.36) | |
| Female |  |  |  |  |  | |
| < 30 | 1,100,895 | 193,434 | 10,270,086 | 18.83 | 1(Ref.) | |
| 30-59 | 314,043 | 60,805 | 2,901,642 | 20.96 | 1.04 (1.03, 1.05) | |
| ≥ 60 | 82,682 | 15,781 | 761,223 | 20.73 | 1.06 (1.04, 1.08) | |

Abbreviations: p-y, person-year; HR, hazard ratio; CI, confidence interval

*Adjusted for age, sex, body mass index, income, smoking, alcohol consumption, regular exercise, hypertension, and dyslipidemia

**Supplementary Table 3. Stratified analyses of the association between fatty liver index and incident fracture by sex:** Hazard ratio (95% Confidence Interval) for incident fracture.

| Category | Body mass index  (kg/m^2^) | Fatty liver index | | | *P* for interaction |
| --- | --- | --- | --- | --- | --- |
|  |  | < 30 | 30-59 | ≥ 60 |  |
| Male | < 25 | 1 (Ref.) | 1.09 (1.07,1.11) | 1.34 (1.31,1.38) | < 0.001 |
|  | ≥ 25 | 1 (Ref.) | 1.03 (1.00,1.07) | 1.22 (1.18,1.26) |  |
| Female | < 25 | 1 (Ref.) | 1.05 (1.04,1.07) | 1.16 (1.11,1.22) | < 0.001 |
|  | ≥ 25 | 1 (Ref.) | 1.01 (1.00,1.02) | 1.04 (1.05,1.06) |  |

Adjusted for age, body mass index, income, smoking, alcohol consumption, regular exercise, hypertension, diabetes, and dyslipidemia
